# Cardiovascular and microvascular outcomes of glucagon-like peptide-1 receptor agonists in type 2 diabetes: a meta-analysis of randomized controlled cardiovascular outcome trials with trial sequential analysis

**DOI:** 10.1186/s40360-018-0246-x

**Published:** 2018-09-17

**Authors:** Xiaowen Zhang, Fei Shao, Lin Zhu, Yuyang Ze, Dalong Zhu, Yan Bi

**Affiliations:** 1 Department of Endocrinology, Affiliated Drum Tower Hospital, Nanjing University School of Medicine, 321 Zhongshan Road, Nanjing, Jiangsu Province, 210008 China; 2 Department of Endocrinology, Nanjing Drum Tower Hospital Clinical College of Traditional Chinese and Western Medicine, Nanjing University of Chinese Medicine, 321 Zhongshan Road, Nanjing, Jiangsu Province, 210008 China

**Keywords:** Glucagon-like peptide-1 receptor agonist, Cardiovascular outcome, Microvascular outcome, Meta-analysis, Trial sequential analysis, Randomized controlled trial

## Abstract

**Background:**

Efficacy trials showed that glucagon-like peptide–1 receptor (GLP1R) agonists reduced metabolic risk factors in addition to glucose lowering, but the cardiovascular and microvascular efficacy of this drug class remains to be determined. We aimed to evaluate the overall cardiovascular and microvascular efficacy of GLP1R agonists by performing a meta-analysis with trial sequential analysis.

**Methods:**

Randomized controlled, cardiovascular outcomes trials including at least 2000 patient-years’ follow-up and 100 composite cardiovascular events were included. Trial sequential analysis (TSA) was performed and the quality of evidence was graded.

**Results:**

Thirty-three thousand four hundred fifty-seven patients and 4105 cardiovascular events from 4 large trials were included. GLP1R agonists were associated with a statistically significant reduction in risks for all-cause mortality (hazard ratio [HR]: 0.88, 95% CI: 0.81 to 0.95; number needed to treat [NNT]: 286 person-years), cardiovascular mortality (HR: 0.87, 95% CI: 0.79 to 0.96; NNT: 412 person-years), stroke (HR: 0.87, 95% CI: 0.76 to 0.98; NNT: 209 person-years) and the composite adverse cardiovascular outcome (MACE; HR: 0.91, 95% CI: 0.85 to 0.96; NNT: 241 person-years). The magnitude of benefit on MACE was attenuated in patients with a history of congestive heart failure (HR: 0.96, 95% CI: 0.85 to 1.08 with; HR: 0.87, 95% CI: 0.77 to 1.00 without). The risks for hospitalization for heart failure and myocardial infarction were not significantly different. The quality of the evidence was deemed as moderate to high based on GRADE approach. TSA provided firm evidence for a 10% reduction in all-cause mortality, a 15% reduction in MACE, and lack of a 15% reduction in hospitalization for heart failure, but evidence remains inconclusive for cardiovascular mortality and myocardial infarction. GLP1R agonists numerically reduced the rates for nephropathy but the risk for retinopathy was similar.

**Conclusions:**

Meta-analysis with trial sequential analysis suggested that GLP1R agonists significantly reduced the risk for all-cause mortality and composite cardiovascular outcomes, but the reduction of cardiovascular mortality remains to be confirmed.

**Electronic supplementary material:**

The online version of this article (10.1186/s40360-018-0246-x) contains supplementary material, which is available to authorized users.

## Background

Type 2 diabetes mellitus (T2DM) is complex metabolic disorder associated with an increased risk for cardiovascular, microvascular and other complications [1]. The promising glucose-lowering effects as well the microvascular benefits of many anti-hyperglycemic drugs have been well documented, but the cardiovascular benefits of these drugs are uncertain [2–4]. Some hypoglycemic medications may even increase rather than reduce the risk of cardiovascular events [5, 6]. Consequently, the US Food and Drug Administration (FDA) have mandated cardiovascular safety assessments of new diabetes treatments [7, 8]. Incretin-based therapies, which include glucagon-like peptide–1 receptor (GLP1R) agonists and dipeptidyl peptidase–4 inhibitors (DDP-4i), are one of the many new anti-hyperglycemic drugs since the last decade. They have now emerged as popular treatment option for glycemic control because of their excellent performance in reducing body weight, blood pressure and postprandial lipoproteins, in addition to marked glucose lowering [9–11]. Whether these metabolic benefits might translate into cardiovascular and microvascular benefits remains an important issue. Several large-scale randomized controlled trials (RCTs) have addressed this issue but the conclusions were not consistent. In this context, we performed a meta-analysis of randomized controlled trials to investigate the effect of GLP1R agonists on cardiovascular outcomes. We also performed trial sequential analysis (TSA) to reduce type I error in meta-analysis to evaluate whether findings from meta-analyses were conclusive or not.

## Methods

We conducted the meta-analysis in accordance with the Preferred Reporting Items for Systematic Reviews and Meta-Analyses (PRISMA) (Additional file 1) and Meta-analysis Of Observational Studies in Epidemiology (MOOSE) guidelines (Additional file 2) [12].

### Data sources and searches

We searched MEDLINE, the Cochrane Central Register of Controlled Trials, and EMBASE from their inception to 28 September 2017 without language restrictions. The following keywords were used: glucagon-like-peptide-1 receptor agonists, exenatide, liraglutide, dulaglutide, albiglutide, lixisenatide, semaglutide, taspoglutide, and randomized controlled trial. Reference lists of the identified reports and relevant reviews were also checked for further relevant studies.

### Study selection

Two reviewers independently assessed the eligibility of studies. To be included, studies had to 1) be randomized controlled, cardiovascular outcome trials which made direct comparisons of glucagon-like-peptide-1 receptor agonists with placebo or active antidiabetic drugs of other classes; 2) contain at least 2000 patient-years of follow-up and 100 composite cardiovascular events to exclude small trials with unreliable hazard ratios; 3) have cardiovascular outcomes predefined and independently adjudicated; and 4) report at least one of our selected cardiovascular outcomes. Discrepancies, if any, were resolved by consensus by a third independent investigator.

### Outcome measures

The primary endpoints were all-cause and cardiovascular death. Secondary endpoints were myocardial infarction, stroke, hospitalization for heart failure, and the major adverse cardiovascular event (MACE) defined as the composite of death from cardiovascular causes, nonfatal myocardial infarction or nonfatal stroke. Microvascular outcomes included nephropathy and retinopathy. We also included efficacy outcomes in the analysis.

### Data extraction and quality assessment

Data extraction was performed by two investigators from each trial using clearly defined extraction forms. The following items were recorded: the registry number, type of treatments, number of patients, follow-up duration, patient demographic and clinical data (age, sex, duration of diabetes, baseline HbA1c levels etc.), history of cardiovascular disease (coronary artery disease, heart failure, stroke, and peripheral arterial disease), medications for diabetes and cardiovascular diseases. We also extracted the number of patients with events and the reported hazard ratio of each outcome. The same reviewers independently assessed the quality of each randomized trial according to the Cochrane Collaboration guideline [13].

### Grading of evidence

We graded the overall methodological quality of each pooled analysis using the Grading of Recommendations, Assessment, Development and Evaluation (GRADE) approach. The quality of evidence was judged as high, moderate, low or very low, using GRADEpro version 3.6 (GRADEpro GDT).

### Data synthesis and statistical analysis

Results of meta-analyses are presented as pooled hazard ratios with their corresponding 95% confidence intervals (CIs). In cases hazard ratios were not available, we used odds ratio instead. We estimated the amount of between-study heterogeneity with the *I*-square statistic and the χ2-based Q test [14]. Data were pooled with the fixed-effects model (Mantel-Haenszel method) because the absence of significant heterogeneity across studies, with random-effect models (DerSimonian–Laird method) as complement [15]. The number needed to treat (NNT) was calculated from randomized trials for risk estimates where risk difference was significant, taking into account the exposure time of treatment within each study. Publication bias was evaluated by funnel plots and Egger’s tests but these tests had limited ability to adequately assess small-study effects because all involved a small number of trials. Predefined subgroup data for MACE based on a variety of clinical variables were directly extracted from each trial and pooled subgroup analyses were conducted. We did a meta-regression analysis to estimate the effects of several covariates—age, gender, body mass index, HbA1c, duration of diabetes, and the percentage of coronary heart disease, chronic heart failure, chronic kidney disease—might have on endpoints with significant association. For the effect estimate, a 2-tailed *p* value less than 0.05 was considered statistically significant. Analyses were done by using the Stata software, version 12.0 (STATA Corporation, College Station, TX, USA).

TSA could reduce type I error because it combines estimation of required information size with adjusted threshold for statistical significance [16–18]. The required information size was calculated based on a relative risk reduction of 10% in all-cause and cardiovascular mortality and a relative risk reduction of 15% in other outcomes. An overall 5% risk of type I error and a statistical test power of 80% were employed.

## Results

### Study selection and characteristics

Of 1980 citations initially identified, 71 were retrieved for full-text evaluation and 4 trials (ELIXA, LEADER, SUSTAIN 6, and EXSCEL) met inclusion criteria (Additional file 3: Figure S1) [19–22]. A total of 33,457 patients receiving GLP1R agonists (*n* = 16,706) or placebo (*n* = 16,751) were included in the analysis. All 4 trials were large, prospective, multicenter, double-blind, randomized placebo-controlled trials with independent cardiovascular endpoint adjudication and adequate follow-up time (range 6924–47,206 patient-years of follow-up). All trials were performed across multiple countries. The mean age enrolled in all the included trials ranged from 56 to 65 years, the mean duration of diabetes from 9.3 to 13.9 years, the mean HbA1c level from 7.7 to 8.7%, and the mean body mass index ranged from 30.2 to 32.8. Overall, 64% patients were male, 70% had a history of coronary heart disease, 18% had heart failure and 23.5% had chronic kidney disease. 46% patients had concomitant insulin use, 74.4% had metformin, 40% had sulfonylureas; new anti-diabetic drugs such as DDP4i and sodium-glucose cotransporter-2 inhibitors were used in very limited number of patients. Cardiovascular medications and other detailed baseline characteristics of each trial are presented in Table 1. Primary and secondary endpoints, inclusion and exclusion criteria of included randomized controlled trials were presented in (Additional file 3: Table S1).

**Table 1:** Characteristics of included randomized controlled trials

Trial	ELIXA	LEADER	SUSTAIN 6	EXSCEL
Year	2015	2016	2016	2017
No. of patients	6068	9340	3297	14,752
No. of countries	49	32	20	35
No. of study sites	NA	310	230	687
Median duration of follow-up, years	2.1	3.8	2.1	3.2
Median duration of exposure to treatment, years	1.9	3.5	1.8	2.4
GLP1R agonist	Lixisenatide	Liraglutide	Semaglutide	Exenatide
Control	Placebo	Placebo	Placebo	Placebo
Age, years	60.3	64.3	64.6	56.0
Male, %	69.3	64.3	60.7	62.0
Diabetes duration, years	9.3	12.7	13.9	12.0
Body mass index	30.2	32.5	32.8	31.7
Current smoking, %	11.7	NA	NA	11.6
HbA1c, %	7.7	8.7	8.7	8.0
Systolic blood pressure, mmHg	129 ± 17	135.9 ± 17.8	135.6 ± 17.2	NA
Diastolic blood pressure, mmHg	NA	77.2 ± 10.3	77.0 ± 10.0	NA
LDL cholesterol, mg/dL	78.8 ± 35.4	NA	82.3 ± 45.6	NA
History of cardiovascular disease				
Hypertension, %	76.4	NA	92.8	NA
Coronary artery disease, %	100	81.4	60.4	52.9
Heart failure, %	22.4	17.9	24.3	16.6
Stroke, %	5.5	NA	12.4	17.3
Peripheral arterial disease, %	7.7	NA	NA	18.9
Chronic kidney disease, %	23.2	24.7	28.5	21.7
Diabetes medications				
Insulin, %	39.1	44.6	58.0	46.4
Metformin, %	66.3	76.4	73.2	76.8
Sulfonylureas, %	33.2	50.5	42.8	36.6
Thiazolidinediones, %	1.6	6.2	2.3	3.9
DPP-4 inhibitor, %	NA	< 0.1	0.2	15.0
Cardiovascular Medications				
ACEI or ARB, %	85.0	82.8	83.5	80.0
Statin, %	92.7	72.1	72.8	73.5
Anti-thrombotic, %	97.5	74.3	76.3	73.6
Beta-blocker, %	84.5	55.4	57.4	55.8
Aldosterone antagonists, %	NA	5.4	5.9	6.2
NCT No.	NCT01147250	NCT01179048	NCT01720446	NCT02098395

*ACEI* angiotensin-converting-enzyme inhibitor, *ARB* angiotensin receptor antagonist, *GLP1R* glucagon-like peptide 1–receptor agonist, *LDL* cholesterol: low-density lipoprotein cholesterol, *NA* not available

The method of random sequence generation was reported in all trials. All trials had blinding of personnel and participants. In 2 trials, blinded outcome adjudication was not performed, so they were judged as being of unclear risk of bias. The risk for detection bias, attrition bias, reporting bias and other bias were generally low. All 4 trials were deemed as good quality, with detailed quality assessment summarized in Additional file 3: Table S2).

### Total and cardiovascular mortality

**Fig. 1:**
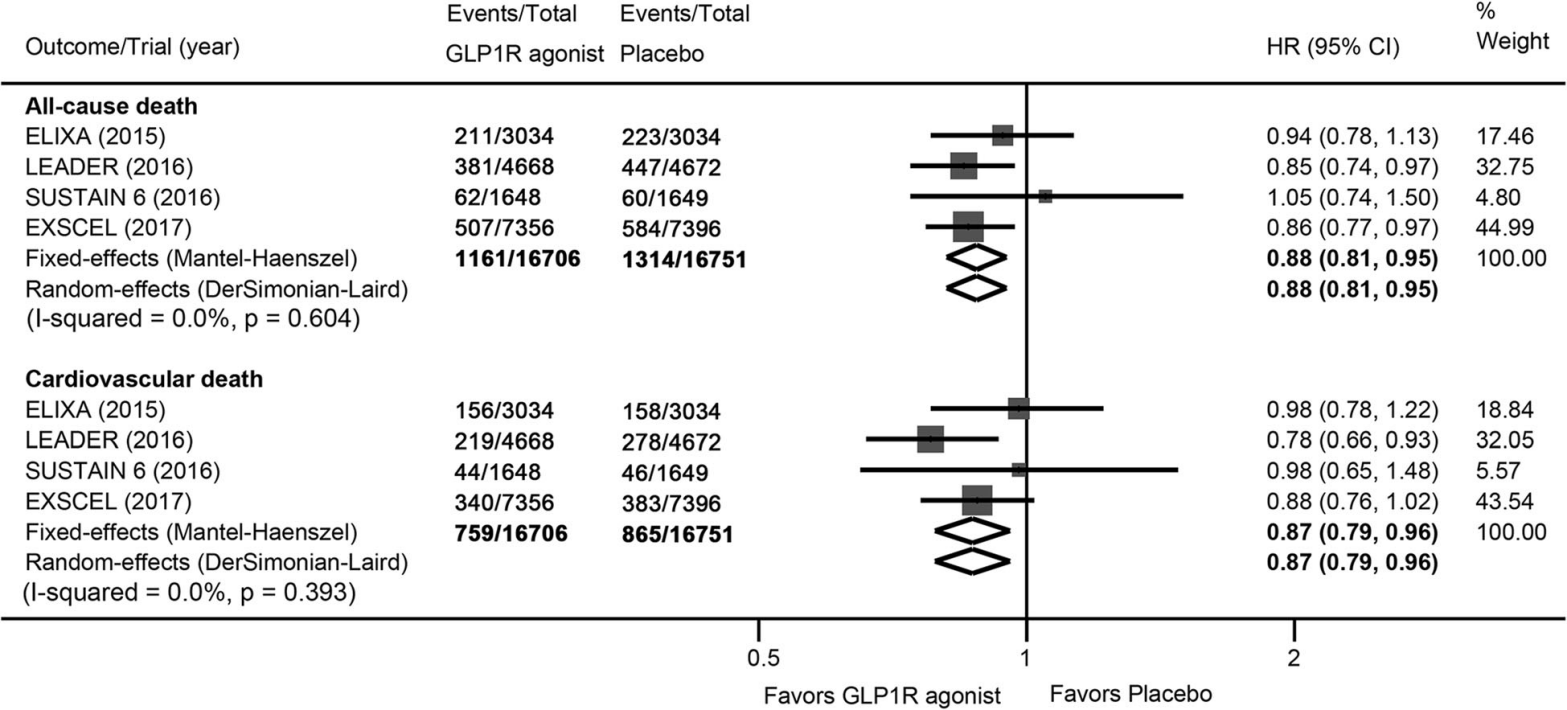
Effects of GLP1R agonists on all-cause and cardiovascular death. CI, confidence interval; GLP1R, glucagon-like peptide–1 receptor; HR, hazard ratio

**Fig. 2:**
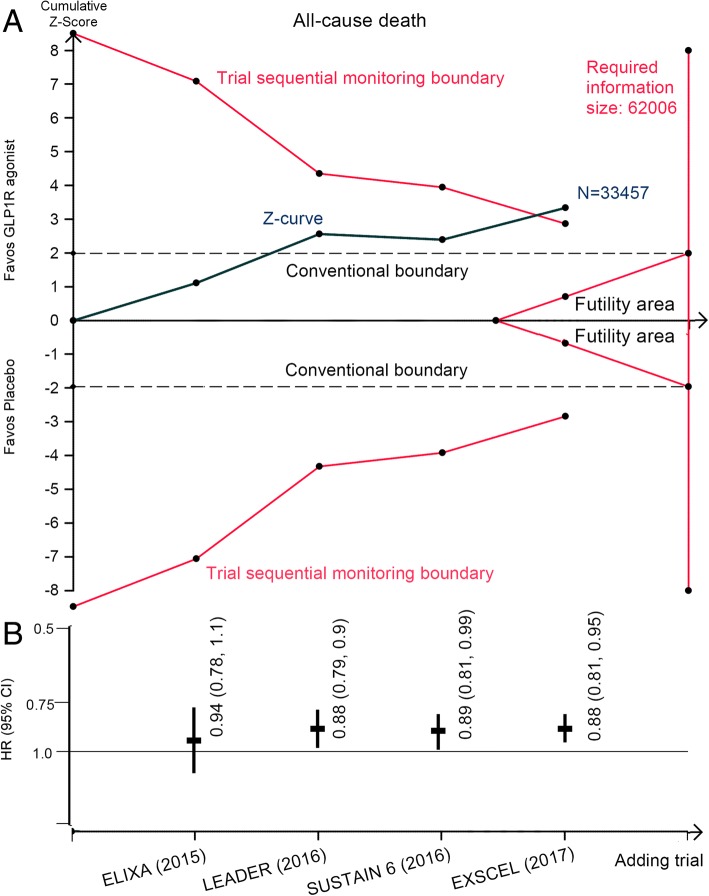
**a** Trial sequential analysis for all-cause death in patients receiving GLP1R agonists versus placebo; **b** Funnel plots showing the trajectory of the overall point estimates and their corresponding precision evolve as each study enter the meta-analysis. CI, confidence interval; GLP1R, glucagon-like peptide–1 receptor; HR, hazard ratio

There were 1161 deaths among 16,706 patients randomly assigned to receive GLP1R agonists and 1314 deaths among 16,751 patients assigned to placebo. Pooled analysis showed a statistically significant reduction in all-cause mortality with GLP1R agonists compared with placebo (HR: 0.88, 95% CI: 0.81 to 0.95, *p* = 0.001; Fig. 1); the NNT was 286 person-years’ exposure to treatment. No evidence of significant heterogeneity was found (*I*^*2*^ = 0). TSA showed that although the pooled sample size did not exceed the estimated required information size, the cumulative Z-curve crossed the conventional boundary and also the trial sequential monitoring boundary (Fig. 2), providing firm evidence for a 10% reduction in total mortality with GLP1R agonists when compared with placebo.

**Fig. 3:**
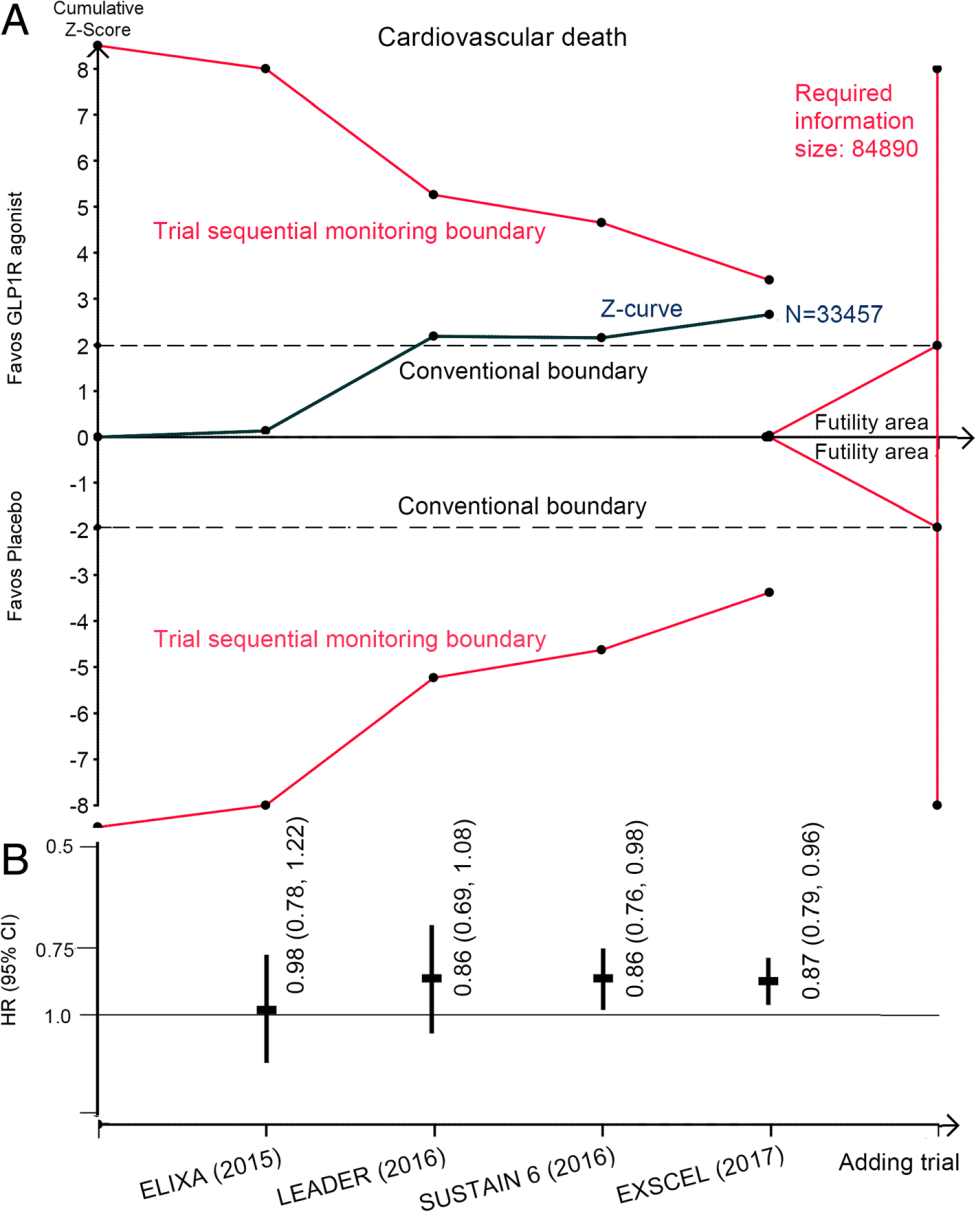
**a** Trial sequential analysis for cardiovascular death in patients receiving GLP1R agonists versus placebo; **b** Funnel plots showing the trajectory of the overall point estimates and their corresponding precision evolve as each study enter the meta-analysis. CI, confidence interval; GLP1R, glucagon-like peptide–1 receptor; HR, hazard ratio

Of 16,706 patients assigned to GLP1R agonists, 759 experienced cardiovascular death, as did 865 of 16,751 patients with placebo. Overall, there was a statistically significant reduction in cardiovascular mortality with use of GLP1R agonists (HR: 0.87, 95% CI: 0.79 to 0.96, *P* = 0.005) (Fig. 1); the number needed to treat was 412 person-years’ exposure to treatment. Similarly, no heterogeneity was detected (*I*^*2*^ = 0). In TSA, the Z-curve crossed the conventional boundary but did not cross the monitoring boundary, indicating a 10% reduction in cardiovascular mortality with GLP1R agonists was inconclusive and that additional evidence is needed (Fig. 3). The risk for non-cardiovascular death was not significantly different (HR: 0.90, 95% CI: 0.78 to 1.03, *P* = 0.11).

### Myocardial infarction and stroke

**Fig. 4:**
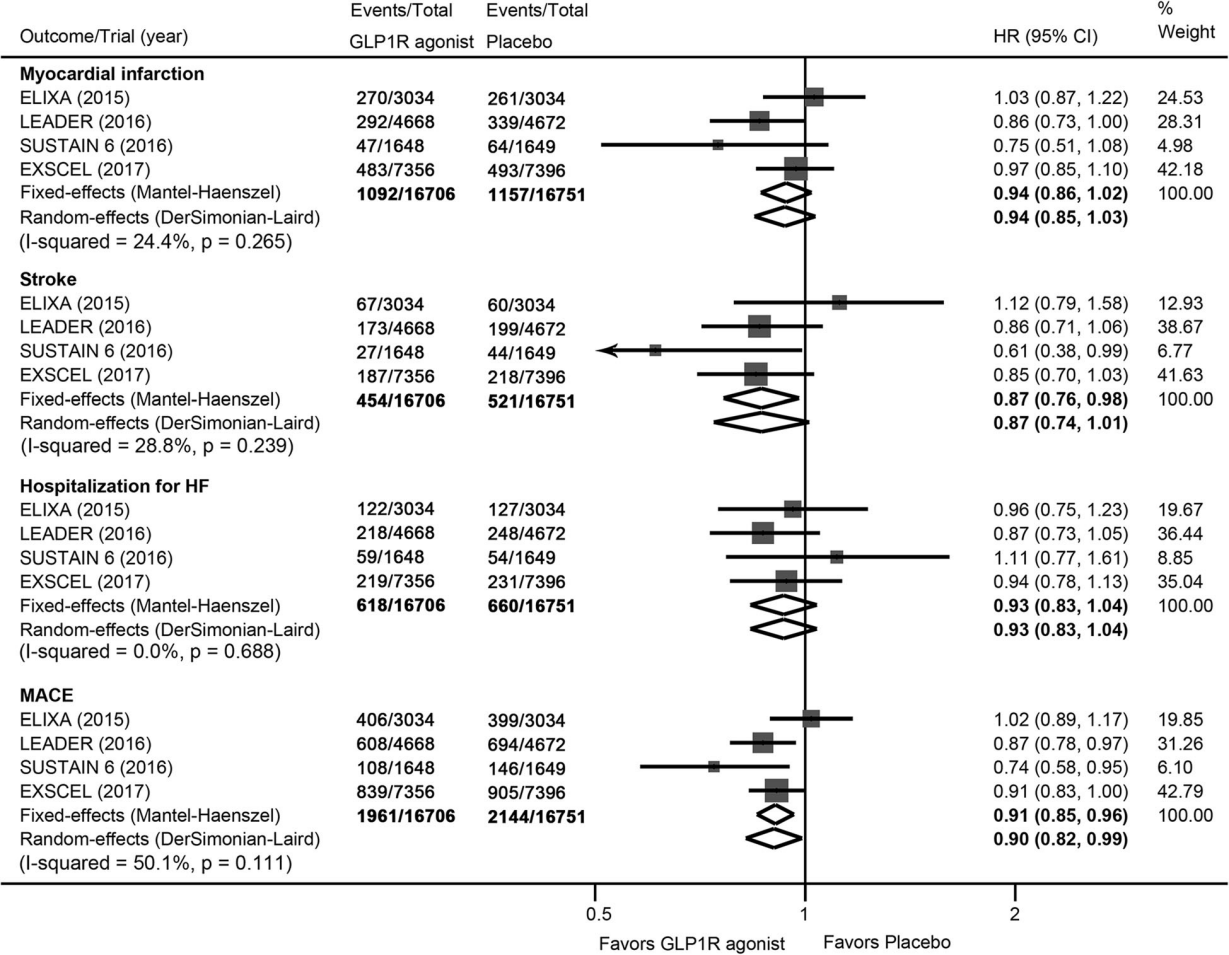
Effects of GLP1R agonists on myocardial infarction, stroke, hospitalization for heart failure, and MACE. CI, confidence interval; GLP1R, glucagon-like peptide–1 receptor; HR, hazard ratio; MACE: major adverse cardiovascular event

Meta-analysis did not show statistically significant difference in incidence of myocardial infarction between patients with GLP1R agonists and those with placebo (HR: 0.94, 95% CI: 0.86 to 1.02, *P* = 0.143; Fig. 4). GLP1R agonists were associated with a statistically significant reduction in rates of stroke (HR: 0.87, 95% CI: 0.76 to 0.98, *P* = 0.023; Fig. 4); the number needed to treat was 209 person-years’ exposure to treatment. We did not find significant heterogeneity in both comparisons (*I*^*2*^ = 24.4% and 28.8 respectively). TSA suggested that a 15% reduction in myocardial infarction and stroke was inconclusive and future trials are needed (Additional file 3: Figures S2 and S3).

### Hospitalization for heart failure

There was no significant difference in rate of hospitalization for heart failure between the use of GLP1R agonists and placebo (HR: 0.93, 95% CI: 0.83 to 1.04, *P* = 0.203) (Fig. 4). We did not find evidence of heterogeneity across these trials (*I*^*2*^ = 0). In TSA, the cumulative Z-curve crossed the futility area, suggesting firm evidence for lack of a 15% relative risk reduction in risk for hospitalization for heart failure (Additional file 3: Figure S4).

### Major adverse cardiovascular event

**Fig. 5:**
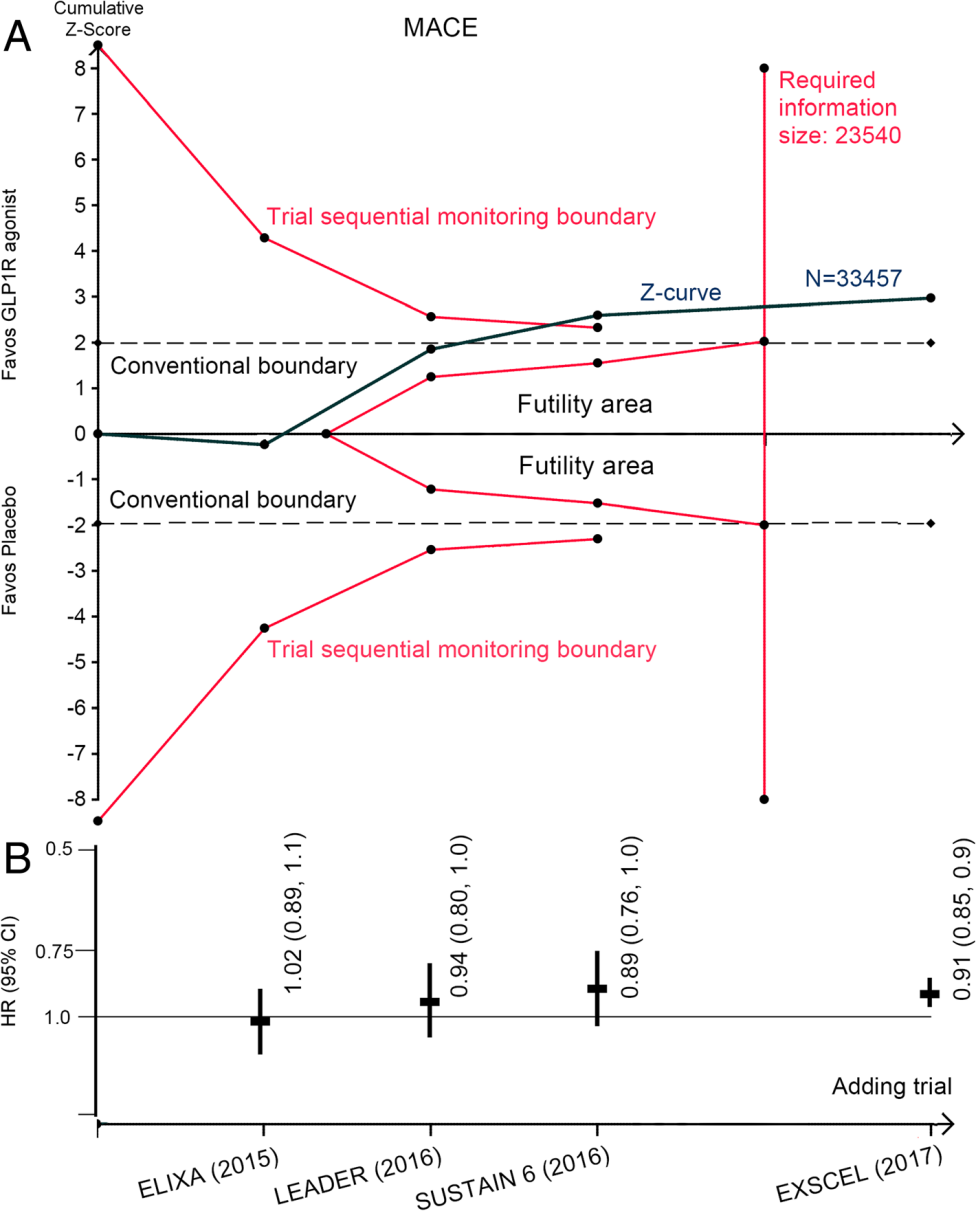
**a** Trial sequential analysis for MACE in patients receiving GLP1R agonists versus placebo; **b** Funnel plots showing the trajectory of the overall point estimates and their corresponding precision evolve as each study enter the meta-analysis. CI, confidence interval; GLP1R, glucagon-like peptide–1 receptor; HR, hazard ratio; MACE: major adverse cardiovascular event

Treatment with GLP1R agonists resulted in a statistically significant reduction in MACE compared with placebo; rates were 11.7% (1961 of 16,706 patients) and 12.8% (2144 of 16,751 patients) respectively (HR: 0.91, 95% CI: 0.85 to 0.96, *P* = 0.002) (Fig. 4). Considerable heterogeneity was detected (*I*^*2*^ = 50.1%); the number needed to treat was 241 person-years’ exposure to treatment. TSA showed that the pooled sample size exceeded the estimated required information size and the cumulative Z-curve crossed both the conventional boundary and the trial sequential monitoring boundary, indicating that there is firm evidence for a 15% reduction in risk for MACE with GLP1R agonists when compared with placebo (Fig. 5).

Largely consistent results on MACE were found across a number of subgroup analyses. The magnitude of the benefit of GLP1R agonists on MACE was attenuated in patients with a younger age, with a body mass index less than 30, with a history of congestive heart failure (Additional file 3: Figure S5), but the tests for interaction were not significant (Table 2).

**Table 2:** Subgroup analyses for MACE

Subgroups	HR (95% CI)	*P* value	*P* value for interaction
Age			
< 60–65 years	0.92 (0.77, 1.10)	0.38	0.58
≥ 60–65 years	0.85 (0.79, 0.93)	0.001	
Gender			
Male	0.91 (0.85, 0.98)	0.02	0.53
Female	0.87 (0.78, 0.98)	0.02	
Race			
White	0.94 (0.88, 1.01)	0.09	0.12
Black	0.77 (0.59, 1.00)	0.05	
Asian	0.78 (0.62, 0.97)	0.03	
Body mass index			
< 30 kg/m^2^	0.93 (0.83, 1.05)	0.22	0.69
≥ 30 kg/m^2^	0.88 (0.81, 0.96)	0.008	
Glycated hemoglobin level			
< 8.0–8.5%	0.91 (0.83, 1.00)	0.04	0.73
≥ 8.0–8.5%	0.89 (0.82, 0.97)	0.01	
Insulin therapy			
Yes	0.88 (0.81, 0.97)	0.01	0.98
No	0.88 (0.80, 0.98)	0.01	
Duration of diabetes			
< 10–15 years	0.91 (0.81, 1.02)	0.09	0.74
≥ 10–15 years	0.89 (0.82, 0.96)	< 0.01	
History of congestive heart failure			
Yes	0.96 (0.85, 1.08)	0.47	0.27
No	0.87 (0.77, 1.00)	0.05	
Estimated GFR			
≥ 60 mL/min/1.73m^2^	0.91 (0.79, 1.05)	0.19	0.82
< 60 mL/min/1.73m^2^	0.89 (0.72, 1.09)	0.25	
≥ 30 mL/min/1.73m^2^	0.88 (0.82, 0.95)	< 0.001	0.81
< 30 mL/min/1.73m^2^	0.87 (0.55, 1.39)	0.57	

*CI* confidence interval, *GFR* glomerular filtration rate, *HR* hazard ratio, *MACE* major adverse cardiovascular event

### Microvascular outcomes

**Fig. 6:**
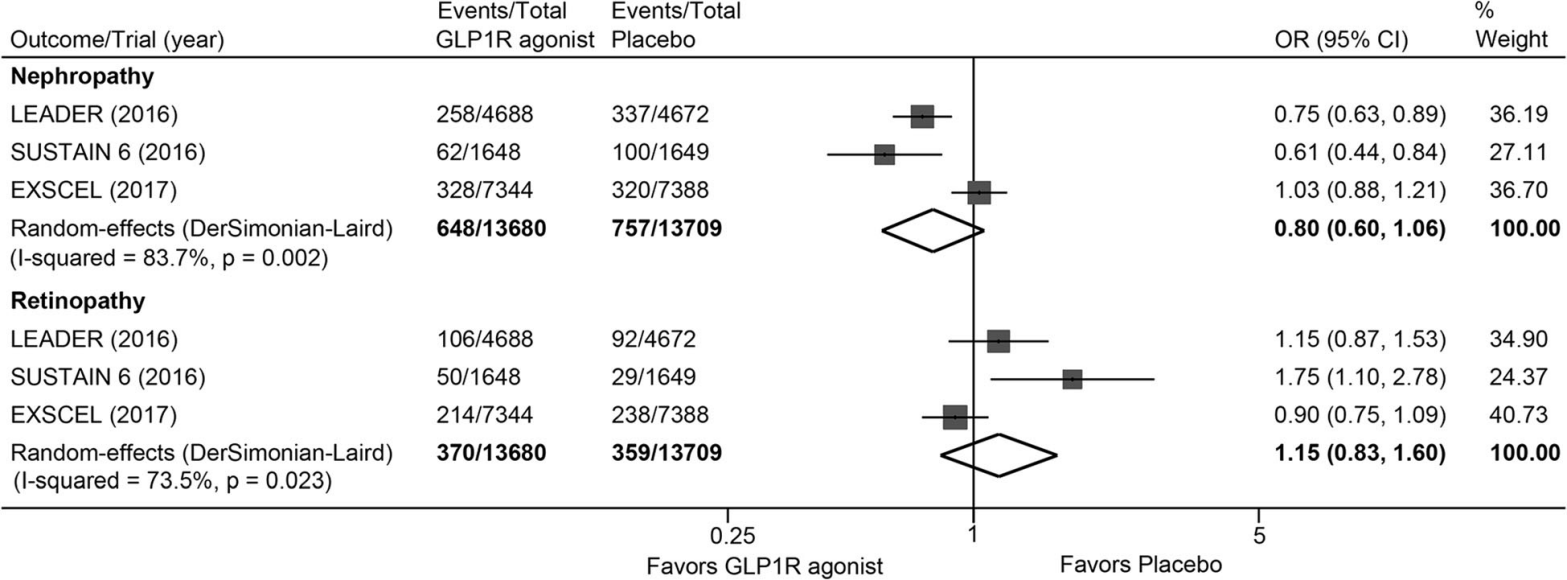
Effects of GLP1R agonists on nephropathy and retinopathy. CI, confidence interval; GLP1R, glucagon-like peptide–1 receptor; OR, odds ratio

The rates for nephropathy was numerically lower in patients with GLP1R agonists than placebo (648/13680 versus 757/13709, OR: 0.80, 95% CI: 0.60 to 1.06, *P* = 0.121) (Fig. 6). There was no significant difference in risk for retinopathy (370/13680 versus 359/13709, OR: 1.15, 95% CI: 0.83 to 1.60, *P* = 0.394) between the use of GLP1R agonists and placebo (Fig. 6). Significant heterogeneity was detected across trials in both analyses (*I*^*2*^ = 83.7% and 73.5% respectively).

### Grading of evidence

Based on GRADE summaries (Table 3), we deemed the quality of the evidence to be high for total and cardiovascular mortality and stroke, and moderate for other outcomes. Reasons for rating down were provided in Table 3.

**Table 3:** GRADE assessment of confidence in estimates of effect in randomized trials

Outcome	No. of participants (trials)	Risk of bias	Consistency	Directness	Precision	Publication bias	Quality
MACE	33,457 (4)	No serious limitations	Serious limitations^a^	No serious limitations	No serious limitations	No serious limitations	Moderate
All-cause death	33,457 (4)	No serious limitations	No serious limitations	No serious limitations	No serious limitations	No serious limitations^d^	High
Cardiovascular death	33,457 (4)	No serious limitations	No serious limitations	No serious limitations	No serious limitations	No serious limitations	High
Myocardial infarction	33,457 (4)	No serious limitations	No serious limitations^b^	No serious limitations	Serious limitations^c^	No serious limitations	Moderate
Stroke	33,457 (4)	No serious limitations	No serious limitations^b^	No serious limitations	No serious limitations	No serious limitations	High
Hospitalization for heart failure	33,457 (4)	No serious limitations	No serious limitations	No serious limitations	Serious limitations^c^	No serious limitations	Moderate

*GRADE* Grading of Recommendations Assessment, Development and Evaluation

^a^Moderate to substantial heterogeneity: I^2^ = 50.1%

^b^I^2^ = 24.4 and 28.8% respectively. Did not downgrade for mild heterogeneity

^c^95% confidence interval (CI) include important harm and benefit

^d^Did not downgrade even though Egger’s test detected a possible publication bias. We did not downgrade because all trials included were large-scale randomized trials and these tests had limited ability to adequately assess small-study effects due to a small number of trials

### Efficacy outcomes

**Fig. 7:**
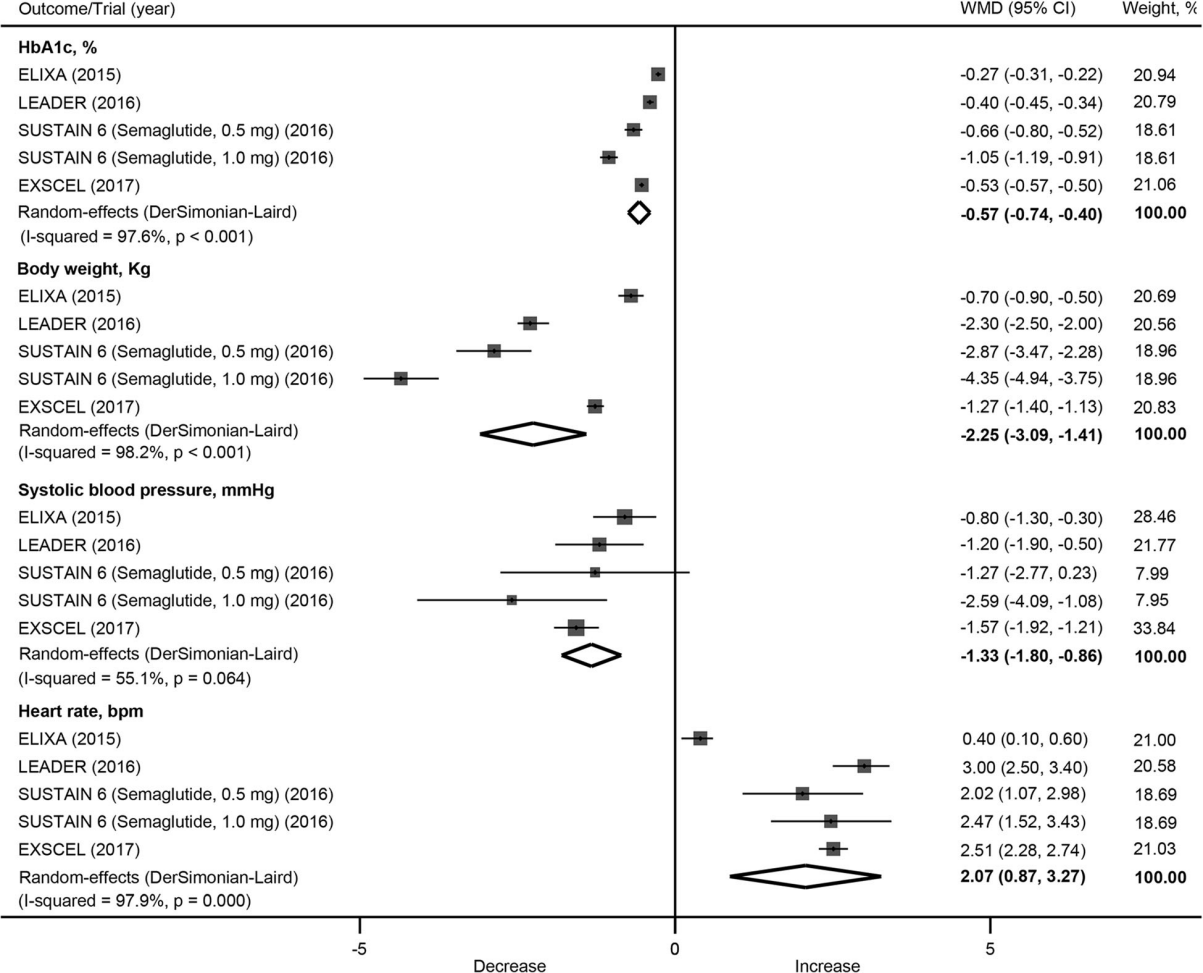
Effects of GLP1R agonists on HbA1c, body weight, systolic blood pressure and heart rate. CI, confidence interval; GLP1R, glucagon-like peptide–1 receptor; WMD, weighted mean difference

GLP1R agonists significantly reduced HbA1c level compared with placebo, with a weighted mean difference of − 0.57% (95% CI: –0.74 to − 0.40) (Fig. 7). GLP1R agonists also significantly reduced body weight (WMD: − 2.25 kg; 95% CI: –3.09 to − 1.41) and systolic blood pressure (WMD: − 1.33 mmHg; 95% CI: –1.80 to − 0.86), and increased heart rate (WMD: 2.07 beats per minute; 95% CI: 0.87 to 3.27).

## Discussion

In this meta-analysis of 33,457 patients and 4105 cardiovascular events from 4 large double-blind, randomized placebo-controlled cardiovascular outcome trials, we showed that 1) GLP1R agonists were associated with a statistically significant reduction in risks for all-cause (high-quality evidence), cardiovascular mortality (high-quality evidence), stroke (high-quality evidence) and the composite outcome of cardiovascular mortality, nonfatal myocardial infarction or nonfatal stroke (moderate-quality evidence); 2) the risks for hospitalization for heart failure and myocardial infarction were not significantly different (moderate-quality evidence); 3) TSA provided firm evidence for a 10% reduction in all-cause mortality, a 15% reduction in MACE, and lack of a 15% reduction in hospitalization for heart failure with GLP1R agonists compared with placebo, but evidence remains inconclusive for cardiovascular mortality and myocardial infarction; 4) the magnitude of the benefit of GLP1R agonists on MACE was more remarkable in patients without a history of congestive heart failure but attenuated in patients with; 5) GLP1R agonists numerically reduced the rates for nephropathy but the risk for retinopathy was similar; 6) GLP1R agonists showed sustained reduction in HbA1c level and a number of other metabolic risk factors.

Several previous meta-analyses have evaluated the cardiovascular effect of GLP1R [23–25]. Two analyses reported no substantial difference in risk for total mortality or the major composite outcomes between GLP1R agonists and control treatments [23, 24]. These analyses were limited to RCTs primarily to evaluate the glucose-lowering efficacy of GLP1R agonists, all with the number of cardiovascular events as few as tens and the number of participants from tens to hundreds. Cardiovascular outcomes in these trials were based on clinician-reported adverse events rather than predefined and independently adjudicated. Another meta-analysis [25] included 3 of the cardiovascular outcome trials. They found that GLP1R agonists were associated with a lower mortality (odds ratio: 0.89; 95% CI: 0.80 to 0.99), but the risk for MACE was not significantly different (odds ratio: 0.88; 95% CI: 0.74 to 1.04). The important EXSCEL trial [22]—the largest trial among the 4 cardiovascular outcome trials was not included in their analysis. Incorporating data from EXSCEL trial to our analysis substantially improved the power of meta-analysis to detect potential difference of rare outcomes (adding another 14,752 patients and 1091 cardiovascular events). Compared with the analysis by Liu and colleagues which only detected total mortality benefit of GLP1R agonists, we extended the benefits to cardiovascular mortality, stroke and the composite MACE. We provided the funnel plots showing the trajectory of the overall point estimates and their corresponding precision evolved as each trial entered the meta-analysis. It can be seen that the benefits on MACE became significant until the EXSCEL trial was included. Another difference was that we employed HR rather than odds ratio in pooled analyses. We chose HR because it allows harmonization of the time-period variability across the trials. Although the direction of results might not be changed using different effect of estimates, the magnitudes of benefit were different. Finally, we determined whether findings from meta-analyses were conclusive by performing TSA and assessed the quality of evidence using the GRADE approach. These comprehensive analyses provide convincing evidence that GLP1R agonists reduce total mortality and MACE.

The US Food and Drug Administration (FDA) requires that all new anti-diabetic drugs must demonstrate good cardiovascular safety with an upper boundary of risk < 1.3. [26] This statement was first raised because a strong and consistent relationship with increased risk of heart failure was reported to be associated with rosiglitazone [5, 6]. Cardiovascular concern has also latter been raised with DDP-4i [27] particularly for saxagliptin [28] also with regard to the risk of admission to hospital for heart failure. Heart failure, one important diabetes complication with high frequency, morbidity and mortality, has previously been relegated to an inferior position compared with microvascular and macrovascular complications in diabetes [4]. Due to this emerging concern, the FDA has now called to have heart failure systematically assessed in cardiovascular outcome trials of all new glucose-lowering drugs [29]. Our analysis, based on > 33,000 patients and 4000 cardiovascular events, provided robust reassurance about the cardiovascular safety of GLP1R agonists, showing no increase in risks for hospitalization for heart failure and myocardial infarction. The upper boundary of risk of each individual and composite outcome was far less than 1.3 in our analysis. Our exploratory finding that the magnitude of the benefit of GLP1R agonists on MACE was more remarkable in patients without a history of congestive heart failure but attenuated in patients with remain to be confirmed in future studies. If confirmed, it would have great impact in the selection of patients for GLP1R agonist use.

Moreover, we found that GLP1R agonists instead reduced the risk for a number of cardiovascular outcomes. In our analysis, a 10% risk reduction in all-cause mortality and a 15% reduction in MACE were evident, although a 10% reduction in cardiovascular mortality still remains inconclusive. The relative risk reduction may seem relatively small, however, it should be duly noted that these benefits were observed in a population with the majority, if not all, patients had established cardiovascular disease (CVD) or at high cardiovascular risk in whom other cardiovascular risk factors were well treated. A large portion of patients were taking concomitant cardiovascular protection medications such as renin–angiotensin–aldosterone system inhibitors, β-blockers, statins or ezetimibe, and acetylsalicylic acid etc. Therefore, the cardiovascular protective effect of GLP1R agonists appears to be additive to that of evidence-based cardiovascular pharmacotherapies [30, 31]. Another important finding of our study was that total mortality was significantly reduced with GLP1R agonists. This finding was considered critically important because the common use of evidence-based cardiovascular pharmacotherapies substantially lower the incidence of mortality, which makes potential difference difficult to detect. For instance, more intensive low-density lipoprotein (LDL) cholesterol–lowering therapy with PCSK9 antibodies or ezetimibe did not show benefit in reducing cardiovascular and total mortality, although a reduced composite outcomes was observed [32–34].

Previous guidelines in cardiology did not make a recommendation on the selection of anti-hyperglycemic drugs in diabetes patients with established CVD or at high cardiovascular risk [30, 31]. The recently published cardiovascular outcomes trials of new antidiabetic therapies have provided additional data on cardiovascular outcomes in patients with type 2 diabetes with cardiovascular disease or at high risk for CVD [19–22, 28, 35–38]. The 2018 Standards of Medical Care in Diabetes recommends that in this population, anti-hyperglycemic therapy should begin with metformin and subsequently incorporate an agent proven to reduce major adverse cardiovascular events and cardiovascular mortality (currently empagliflozin and liraglutide) [39]. Our analyses support the recommendation that anti-hyperglycemic drugs should be chosen with preference in diabetes patients with established CVD or at high cardiovascular risk, with GLP1R agonists and also sodium–glucose cotransporter 2 inhibitors [35] as priority, after considering drug-specific and patient factors.

The mechanisms of cardiovascular protection associated with GLP1R agonists are considered multidimensional, which was discussed elsewhere [40]. It may be associated with reduction of body weight, blood pressure, the lipid profile and lowering the risk for hypoglycemia, and amelioration of insulin resistance and inflammation, etc. Although our findings were obtained in the context of no obvious heterogeneity among trials, whether the cardiovascular benefits of GLP1R agonists represent a class effect remains to be definitively established. Other large-scale randomized controlled cardiovascular outcome trials are ongoing, such as the ITCA 650 trial (NCT01455896) with 4000 patients and the REWIND (Researching Cardiovascular Events With a Weekly INcretin in Diabetes, NCT01394952) with 9622 patients (Additional file 3: Table S3). Of note, overexpression of mTOR stimulated GLP-1 production, indicating a link role of mTOR between energy supply and the production of GLP-1 [41]. Indeed, inhibition of mTOR with rapamycin attenuated inflammation, inhibited progression, and enhanced stability of atherosclerotic plaques in animal models [42]. The potential effect of the inhibition of the mTOR pathway in the treatment of cardiovascular diseases warrants further investigation.

### Limitations

We acknowledge several limitations. First, the results were analyzed on trial level data but not on patient level data; individual patient-level data could improve the accuracy of the findings. Second, our finding that the magnitude of the benefit of GLP1R agonists on MACE was more remarkable in patients without a history of congestive heart failure but attenuated in patients with an only be considered exploratory and remain to be confirmed in future studies. Third, publication bias tests had limited ability to adequately assess small-study effects because all involved a small number of trials. Fourth, the conclusions were based on diabetes patients with established or high risk for CVD and could be generalized to patients with low cardiovascular risk.

## Conclusions

Moderate-to-high quality of evidence suggested that GLP1R agonists did not increase the risk for any of, but instead reduced the risk for a number of cardiovascular outcomes. A 10% reduction in all-cause mortality and a 15% reduction in the composite outcome of cardiovascular death, nonfatal myocardial infarction or nonfatal stroke were evident. In diabetes patients with established CVD or at high cardiovascular risk, GLP1R agonists could be a preferred anti-hyperglycemic agent after considering drug-specific and patient factors. Future studies are needed to confirm the long-term cardiovascular benefits of GLP1R agonists.

## Additional files

Additional file 1: PRISMA checklist. (DOCX 22 kb)

Additional file 2: MOOSE checklist. (DOC 71 kb)

Additional file 3: **Figure S1.** Flow diagram of study selection; **Table S1.** Primary and secondary endpoints, inclusion and exclusion criteria of included randomized controlled trials; **Table S2.** Risk of bias of included randomized controlled trials; **Figure S2.** Trial sequential analysis for myocardial infarction in patients receiving glucagon-like peptide-1 receptor agonists versus placebo; **Figure S3.** Trial sequential analysis for stroke in patients receiving glucagon-like peptide-1 receptor agonists versus placebo; **Figure S4.** Trial sequential analysis for hospitalization for heart failure in patients receiving glucagon-like peptide-1 receptor agonists versus placebo; **Figure S5.** Analysis of MACE based on patients with or without a history of congestive heart failure; **Table S3.** Characteristics of large ongoing randomized controlled trials evaluating cardiovascular efficacy of GLP-1 receptor agonist. (DOCX 1010 kb)

## Abbreviations

CIs: Confidence intervals
CVD: Cardiovascular disease
DDP-4i: Dipeptidyl peptidase–4 inhibitors
FDA: Food and Drug Administration
GLP1R: Glucagon-like peptide–1 receptor
GRADE: Grading of Recommendations, Assessment, Development and Evaluation
HR: Hazard ratio
LDL: Low-density lipoprotein
MACE: Major adverse cardiovascular event
NNT: Number needed to treat
PRISMA: Preferred Reporting Items for Systematic Reviews and Meta-Analyses
RCTs: Randomized controlled trials
T2DM: Type 2 diabetes mellitus
TSA: Trial sequential analysis
