# Supplementary material for: Cardiovascular and microvascular outcomes of glucagon-like peptide-1 receptor agonists in type 2 diabetes: a meta-analysis of randomized controlled cardiovascular outcome trials with trial sequential analysis
Source: BMC Pharmacol Toxicol. 2018 Sep 17;19:58. doi: 10.1186/s40360-018-0246-x (PMC6142638; doi:10.1186/s40360-018-0246-x)
Supplement: Supplementary file 3 — Figure S1. Flow diagram of study selection; Table S1. Primary and secondary endpoints, inclusion and exclusion criteria of included randomized controlled trials; Table S2. Risk of bias of included randomized controlled trials; Figure S2. Trial sequential analysis for myocardial infarction in patients receiving glucagon-like peptide-1 receptor agonists versus placebo; Figure S3. Trial sequential analysis for stroke in patients receiving glucagon-like peptide-1 receptor agonists versus placebo; Figure S4. Trial sequential analysis for hospitalization for heart failure in patients receiving glucagon-like peptide-1 receptor agonists versus placebo; Figure S5. Analysis of MACE based on patients with or without a history of congestive heart failure; Table S3. Characteristics of large ongoing randomized controlled trials evaluating cardiovascular efficacy of GLP-1 receptor agonist. (DOCX 1010 kb) [file 40360_2018_246_MOESM3_ESM.docx]

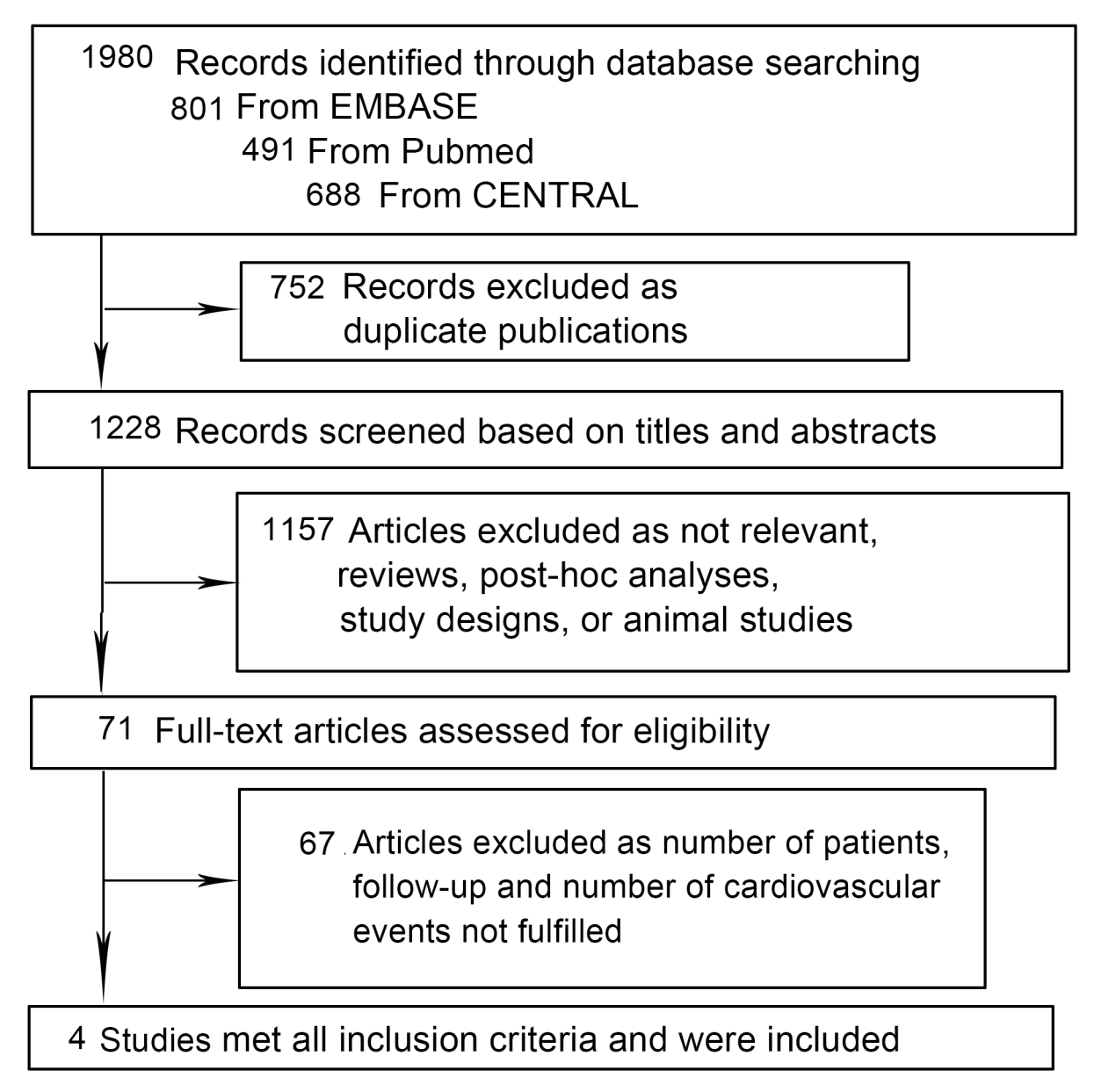


**Figure S1.** Flow diagram of study selection.

**Table S1.** Primary and secondary endpoints, inclusion and exclusion criteria of included randomized controlled trials.

| Trial | Primary endpoint | Secondary endpoint | Inclusion Criteria | Exclusion criteria |
| --- | --- | --- | --- | --- |
| LEADER | Composite of death from cardiovascular causes, nonfatal (including silent) myocardial infarction, or nonfatal stroke. | An expanded composite cardiovascular outcome (death from cardiovascular causes, nonfatal myocardial infarction, nonfatal stroke, coronary revascularization, or hospitalization for unstable angina pectoris or heart failure), death from any cause, a composite renal and retinal microvascular outcome. | Patients with type 2 diabetes who had a glycated hemoglobin level of 7.0% or more, with an age of 50 years or more with at least one cardiovascular coexisting condition (coronary heart disease, cerebrovascular disease, peripheral vascular disease, chronic kidney disease of stage 3 or greater, or chronic heart failure of New York Heart Association class II or III) or an age of 60 years or more with at least one cardiovascular risk factor. | Type 1 diabetes; the use of GLP-1–receptor agonists, dipeptidyl peptidase 4 (DPP-4) inhibitors, pramlintide, or rapid-acting insulin; a familial or personal history of multiple endocrine neoplasia type 2 or medullary thyroid cancer; and the occurrence of an acute coronary or cerebrovascular event within 14 days before screening and randomization. |
| ELIXA | Composite of death from cardiovascular causes, nonfatal myocardial infarction, nonfatal stroke, or hospitalization for unstable angina. | A composite of the primary end point or hospitalization for heart failure and a composite of the primary end point, hospitalization for heart failure, or coronary revascularization procedures. | Patients with type 2 diabetes and had had an acute coronary event within 180 days before screening. | An age of less than 30 years, percutaneous coronary intervention within the previous 15 days, coronary-artery bypass graft surgery for the qualifying event, planned coronary revascularization procedure within 90 days after screening, an estimated glomerular filtration rate (eGFR) of less than 30 ml per minute per 1.73 m2 of body surface area, a glycated hemoglobin level of less than 5.5% or more than 11.0%, or an inability to provide written informed consent. |
| SUSTAIN 6 | The composite of death from cardiovascular causes, nonfatal myocardial infarction (including silent), or nonfatal stroke. | An expanded composite cardiovascular outcome (death from cardiovascular causes, nonfatal myocardial infarction, nonfatal stroke, revascularization [coronary or peripheral], and hospitalization for unstable angina or heart failure), an additional composite outcome (death from all causes, nonfatal myocardial infarction, or nonfatal stroke), the individual components of the composite outcomes, retinopathy complications, and new or worsening nephropathy. | Patients with type 2 diabetes and a glycated hemoglobin level of 7% or more, with an age of 50 years or more with established cardiovascular disease (previous cardiovascular, cerebrovascular, or peripheral vascular disease), chronic heart failure (New York Heart Association class II or III), or chronic kidney disease of stage 3 or higher or an age of 60 years or more with at least one cardiovascular risk factor | Treatment with a dipeptidyl-peptidase 4 inhibitor within 30 days before screening or with a GLP-1–receptor agonist or insulin other than basal or premixed within 90 days before screening; a history of an acute coronary or cerebrovascular event within 90 days before randomization; planned revascularization of a coronary, carotid, or peripheral artery; or long-term dialysis. |
| EXSCEL | The composite outcome of death from cardiovascular causes, nonfatal myocardial infarction, or nonfatal stroke. | Death from any cause, death from cardiovascular causes, and nonfatal or fatal myocardial infarction, nonfatal or fatal stroke, hospitalization for acute coronary syndrome, and hospitalization for heart failure | Adults with type 2 diabetes (defined as a glycated hemoglobin level of 6.5 to 10.0% [48 to 96 mmol per mole]) were eligible for participation in the trial. Approximately 70% of enrolled patients would have had previous cardiovascular events and 30% would not have had previous cardiovascular events. Previous cardiovascular events were defined as a history of major clinical manifestation of coronary artery disease, ischemic cerebrovascular disease, or atherosclerotic peripheral arterial disease. | A history of two or more episodes of severe hypoglycemia during the preceding 12 months, end-stage kidney disease or an estimated glomerular filtration rate (eGFR) at entry of less than 30 ml per minute per 1.73 m2 of body-surface area, a personal or family history of medullary thyroid carcinoma or multiple endocrine neoplasia type 2, a baseline calcitonin level of greater than 40 ng per liter, or previous treatment with a GLP-1 receptor agonist. |

**Table S2.** Risk of bias of included randomized controlled trials.

| Trial | Random sequence generation (selection bias) | Allocation concealment (selection bias) | Blinding of participants and personnel (performance bias) | Blinding of outcome assessment (detection bias) | Incomplete outcome data (attrition bias) | Selective reporting (reporting bias) | Other bias |
| --- | --- | --- | --- | --- | --- | --- | --- |
| LEADER | Low risk | Low risk | Low risk | Low risk | Low risk | Low risk | Low risk |
| ELIXA | Low risk | Low risk | Low risk | Unclear risk | Low risk | Low risk | Low risk |
| SUSTAIN 6 | Low risk | Low risk | Low risk | Unclear risk | Low risk | Low risk | Low risk |
| EXSCEL | Low risk | Low risk | Low risk | Low risk | Low risk | Low risk | Low risk |


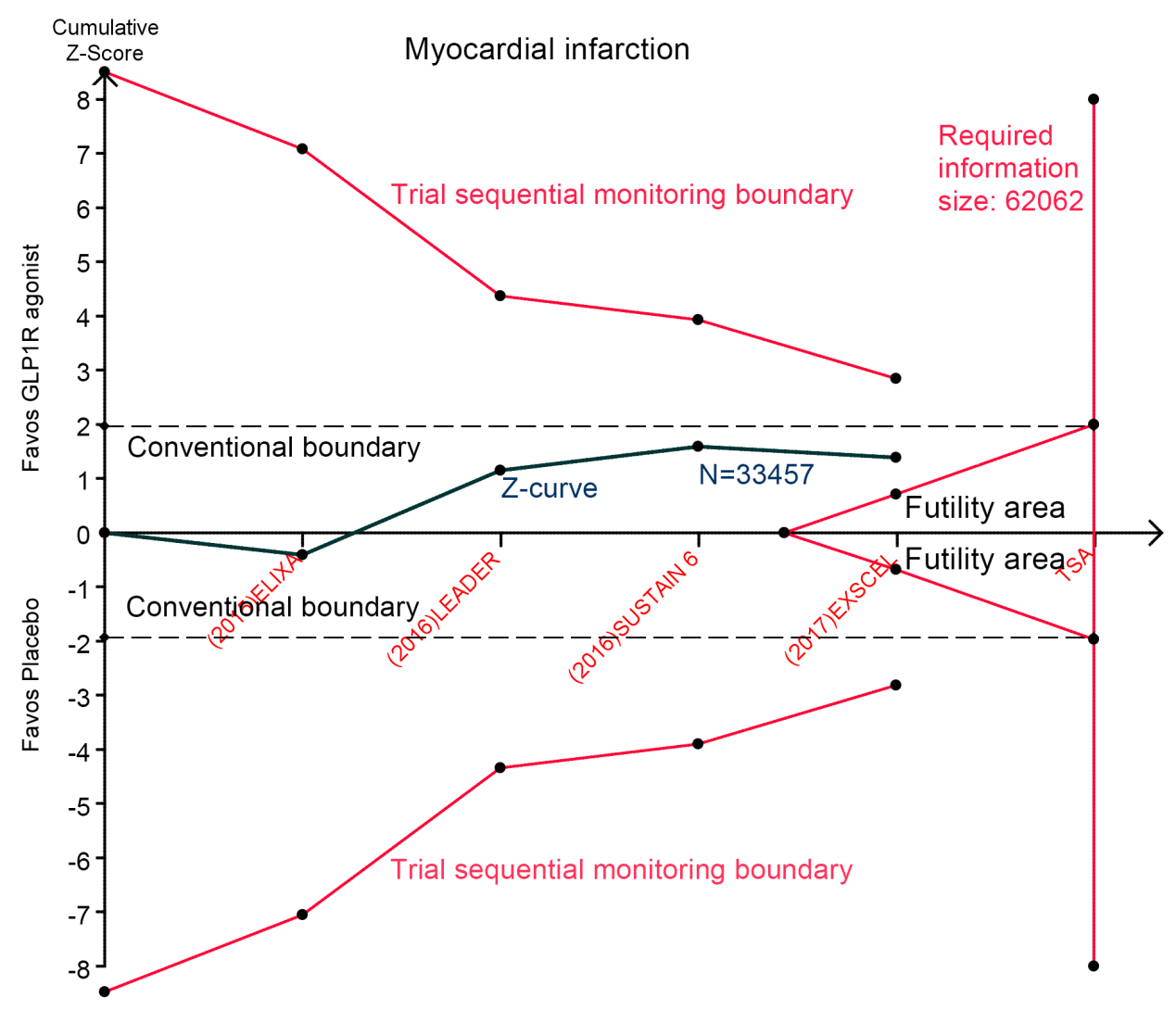


**Figure S2.** Trial sequential analysis for myocardial infarction in patients receiving glucagon-like peptide-1 receptor agonists versus placebo.


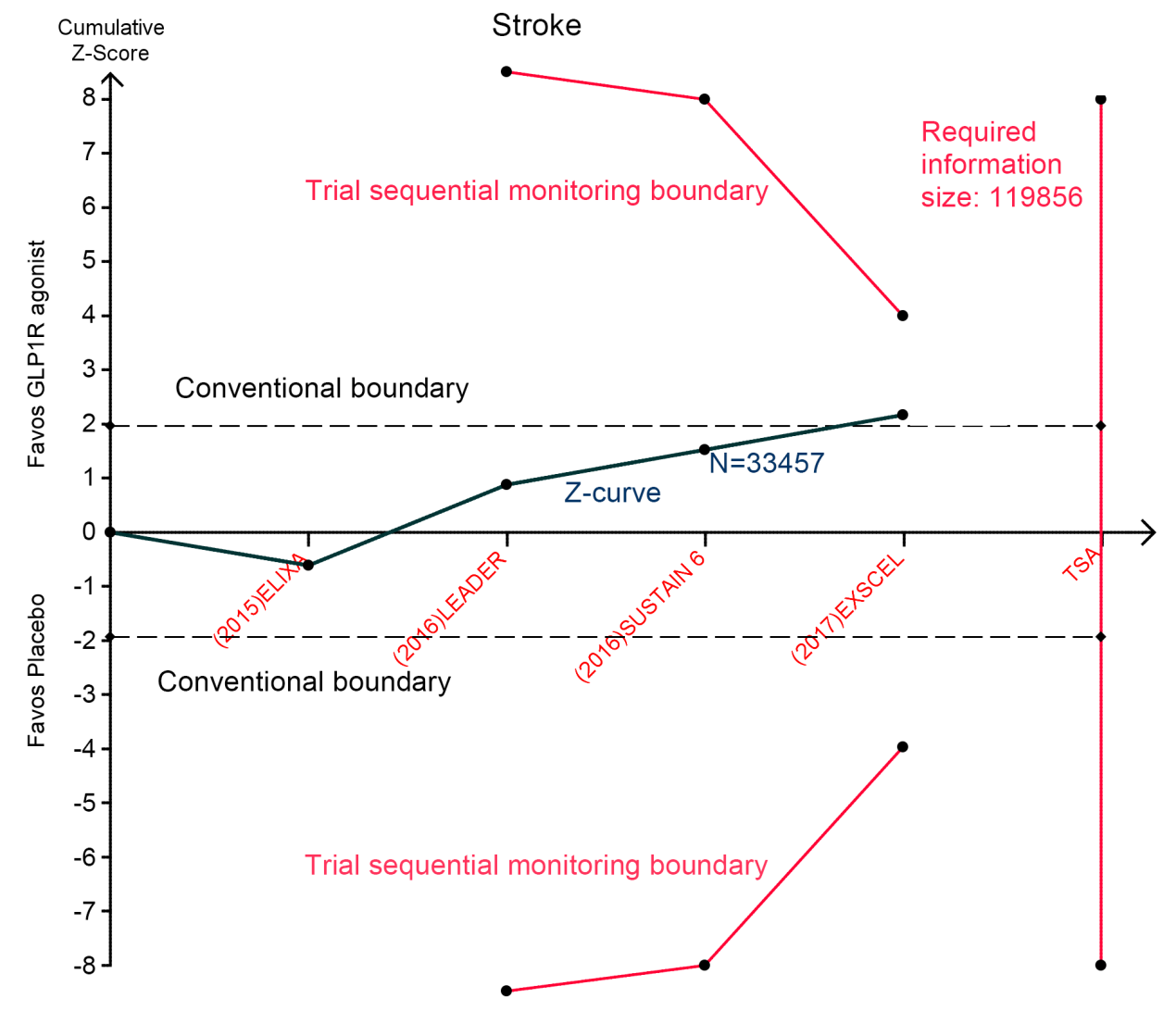


**Figure S3.** Trial sequential analysis for stroke in patients receiving glucagon-like peptide-1 receptor agonists versus placebo.


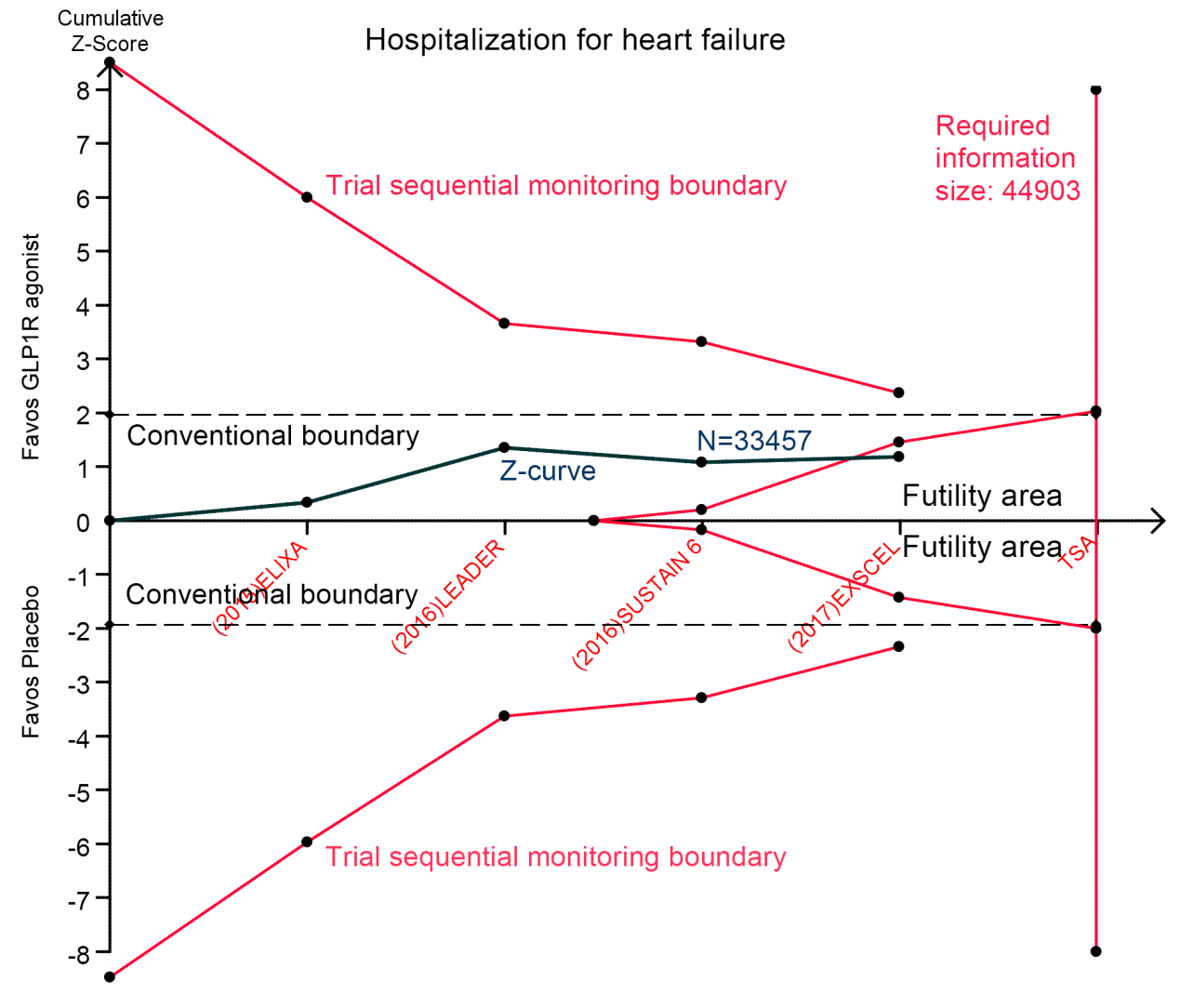


**Figure S4.** Trial sequential analysis for hospitalization for heart failure in patients receiving glucagon-like peptide-1 receptor agonists versus placebo.


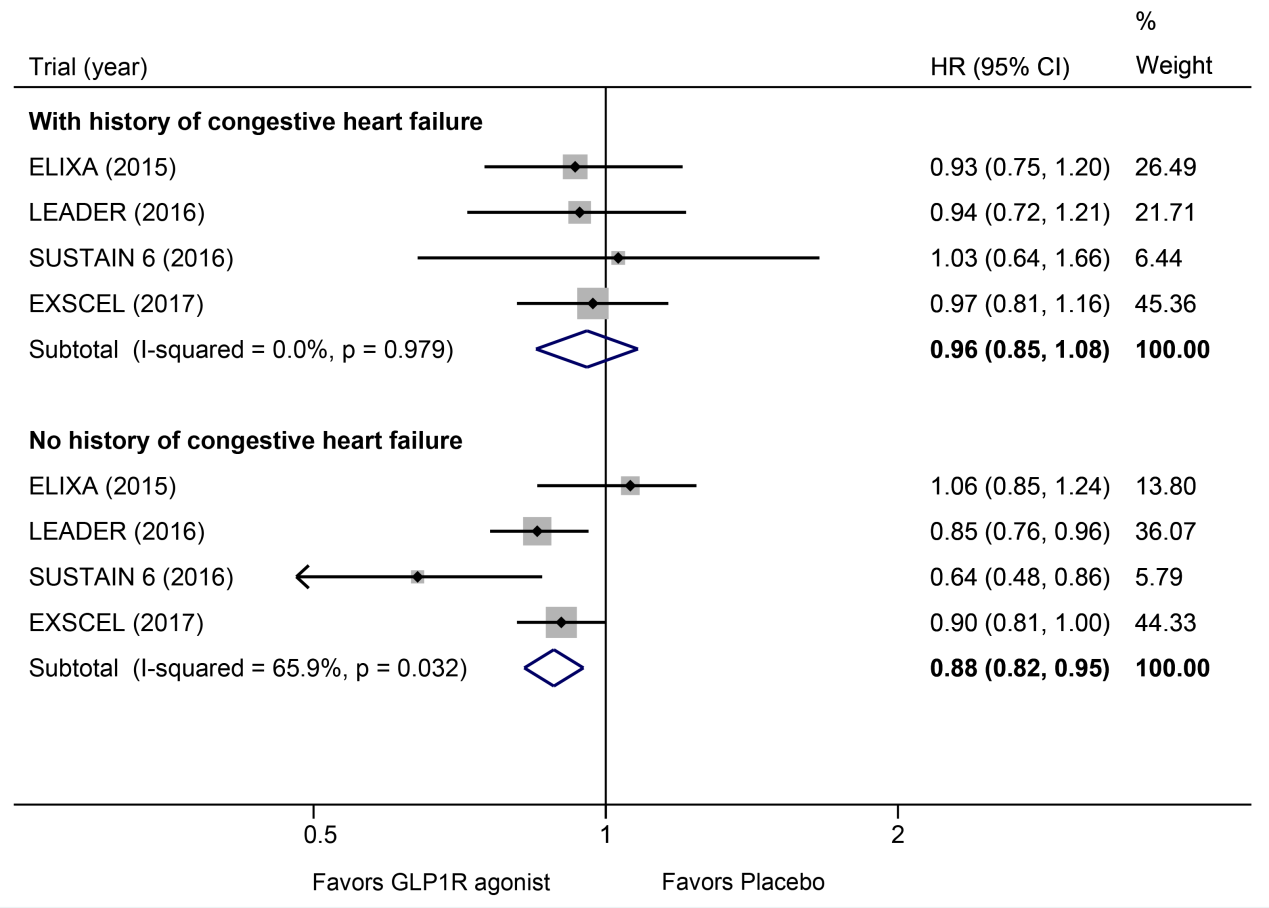


**Figure S5.** Analysis of MACE based on patients with or without a history of congestive heart failure. CI, confidence interval; GLP1R, glucagon-like peptide–1 receptor; HR, hazard ratio.

**Table S3.** Characteristics of large ongoing randomized controlled trials evaluating cardiovascular efficacy of GLP-1 receptor agonist.

| Trial | ITCA 650 | REWIND |
| --- | --- | --- |
| No. of patients | 4000 | 9622 |
| Main inclusion criteria | Type 2 diabetes;  40 Years and older;  HBA1c > 6.5%;  History of coronary, cerebrovascular or peripheral artery disease | Type 2 diabetes with Hemoglobin A1c equal to or less than 9.5%; Age equal to or greater than 50 years with established clinical vascular disease, or age equal to or greater than 55 years and subclinical vascular disease or age equal to or greater than 60 years and at least 2 or more cardiovascular risk factors |
| Main exclusion criteria | History of pancreatitis | Uncontrolled diabetes requiring immediate therapy;  History of severe hypoglycemia in past year;  Acute coronary or cerebrovascular event within past 2 months;  Planned or anticipated revascularization procedure;  History of pancreatitis, hepatic insufficiency , chronic renal failure or of C-cell thyroid disorder;  Pregnancy or planned pregnancy during the trial period;  Completed or withdrawn from any study investigating dulaglutide |
| Intervention | Exenatide versus placebo | Dulaglutide versus placebo |
| Primary endpoint | The composite endpoint of cardiovascular death, nonfatal myocardial infarction, nonfatal stroke, or hospitalization for unstable angina | The composite endpoint of cardiovascular death, nonfatal myocardial infarction, or nonfatal stroke |
| Secondary endpoints | Not available | The composite microvascular endpoint, components of the primary composite endpoint (cardiovascular death, fatal or nonfatal MI, fatal or nonfatal stroke), hospitalization for unstable angina, hospitalization or an urgent heart failure |
| Target follow-up duration | 2 years | 6.5 years |
| Study start date | March 2013 | July 2011 |
| Primary completion date | April 2016 | July 2018 |
| Study completion date | April 2016 | July 2018 |
| ClinicalTrials.gov number | NCT01455896 | NCT01394952 |
| Study design | Prospective, randomized, double-blind, multicenter study | Prospective, randomized, double-blind, multicenter study |

Expanded study abbreviations are as follows: ITCA 650: A Randomized, Multi-Center Study to Evaluate Cardiovascular Outcomes With ITCA 650 in Patients Treated With Standard of Care for Type 2 Diabetes; Researching Cardiovascular Events With a Weekly INcretin in Diabetes (REWIND): The Effect of Dulaglutide on Major Cardiovascular Events in Patients With Type 2 Diabetes.
